# Retinal GABAergic Alterations in Adults with Autism Spectrum Disorder

**DOI:** 10.1523/JNEUROSCI.1218-23.2024

**Published:** 2024-03-11

**Authors:** Qiyun Huang, Claire L. Ellis, Shaun M. Leo, Hester Velthuis, Andreia C. Pereira, Mihail Dimitrov, Francesca M. Ponteduro, Nichol M. L. Wong, Eileen Daly, Declan G. M. Murphy, Omar A. Mahroo, Gráinne M. McAlonan

**Affiliations:** ^1^Department of Forensic and Neurodevelopmental Sciences, Institute of Psychiatry, Psychology & Neuroscience, King’s College London, London SE5 8AF, United Kingdom; ^2^Institute for Translational Neurodevelopment, Institute of Psychiatry, Psychology & Neuroscience, King’s College London, London SE5 8AF, United Kingdom; ^3^Research Center for Brain-Computer Interface, Pazhou Lab, Guangzhou 510665, China; ^4^Moorfields Eye Hospital NHS Foundation Trust, London EC1V 2PD, United Kingdom; ^5^Institute for Nuclear Sciences Applied to Health (ICNAS), Coimbra Institute for Biomedical Imaging and Translational Research (CIBIT), University of Coimbra, Coimbra 3000-548, Portugal; ^6^Department of Psychology, The Education University of Hong Kong, Hong Kong, China; ^7^MRC Centre for Neurodevelopmental Disorders, King’s College London, London SE1 1UL, United Kingdom; ^8^ Institute of Ophthalmology, University College London, London WC1E 6BT, United Kingdom; ^9^Section of Ophthalmology, St Thomas' Hospital, King’s College London, London SE1 7EH, United Kingdom; ^10^Department of Translational Ophthalmology, Wills Eye Hospital, Philadelphia, Pennsylvania 19107

**Keywords:** autism, GABA, retina, sensory processing

## Abstract

Alterations in γ-aminobutyric acid (GABA) have been implicated in sensory differences in individuals with autism spectrum disorder (ASD). Visual signals are initially processed in the retina, and in this study, we explored the hypotheses that the GABA-dependent retinal response to light is altered in individuals with ASD. Light-adapted electroretinograms were recorded from 61 adults (38 males and 23 females; *n* = 22 ASD) in response to three stimulus protocols: (1) the standard white flash, (2) the standard 30 Hz flickering protocol, and (3) the photopic negative response protocol. Participants were administered an oral dose of placebo, 15 or 30 mg of arbaclofen (STX209, GABA_B_ agonist) in a randomized, double-blind, crossover order before the test. At baseline (placebo), the a-wave amplitudes in response to single white flashes were more prominent in ASD, relative to typically developed (TD) participants. Arbaclofen was associated with a decrease in the a-wave amplitude in ASD, but an increase in TD, eliminating the group difference observed at baseline. The extent of this arbaclofen-elicited shift significantly correlated with the arbaclofen-elicited shift in cortical responses to auditory stimuli as measured by using an electroencephalogram in our prior study and with broader autistic traits measured with the autism quotient across the whole cohort. Hence, GABA-dependent differences in retinal light processing in ASD appear to be an accessible component of a wider autistic difference in the central processing of sensory information, which may be upstream of more complex autistic phenotypes.

## Significance Statement

Our current study provides the first direct in vivo experimental confirmation that autistic alterations in central γ-aminobutyric acid (GABA) function extend to the retina. We show that arbaclofen was associated with reduced flash-elicited a-wave amplitude in the electroretinogram (ERG) of autistic individuals but increased amplitude in nonautistic people. The retinal arbaclofen response correlated with previously reported arbaclofen effects on cortical visual and auditory responses in the same individuals. The extent of this differential GABAergic function correlated with the extent of autistic traits captured using the autism quotient. Thus, sensory processing differences in autism appear to be upstream of more complex autistic traits, and the ERG from the retina is a potentially useful proxy for cross-domain brain GABA function and target engagement.

## Introduction

Altered sensory reactivities and interests were recognized in the original descriptions of autism spectrum disorder (ASD) (Kanner, 1951; Asperger, 1944) and are now considered core to its diagnosis (American Psychiatric Association, 2013). Indeed, sensory features are among the first differences reported in infants who go on to receive a diagnosis of ASD and predict the severity of other core behavioral symptoms of ASD, such as social communication deficits and repetitive behaviors (Kern et al., 2007; Boyd et al., 2009; Simmons et al., 2009). These sensory differences span modalities. We and others have reported that individuals with ASD tend not to suppress cortical responses to repeated auditory tones (Kolesnik et al., 2019). In the tactile domain, for example, children with ASD have weaker amplitude discrimination than people with typical development (TD) and show less adaptation (Puts et al., 2014). In the visual domain, stronger focus on local detail (Dakin and Frith, 2005), hypersensitivity to dark or bright lights, dislike of light flashes, and unusual fascination with reflections (Leekam et al., 2007; Bogdashina, 2016) have been reported in individuals with ASD. This has fueled the argument that sensory processing differences in ASD are core, and evidence is emerging that sensory alterations are in fact upstream of the more complex social–cognitive phenotype of ASD (Robertson and Baron-Cohen, 2017). Sensory differences may therefore be a more proximal target for interventions with “knock-on” effects on wider functions.

This is important because, despite the cross-modality nature of findings, it has been suggested that autistic sensory differences are thought to arise from genetic and/or prenatal environmental factors that alter excitatory–inhibitory (E–I) balance (Rubenstein and Merzenich, 2003). Until recently, however, the majority of prior studies in humans have relied on correlational or associative analyses between cortical γ-aminobutyric acid (GABA) levels and/or genes encoding GABA pathway components and sensory function (Robertson et al., 2016; Puts et al., 2017; Hollestein et al., 2023). Correlations, however, are not causal. A more direct way to establish that a particular neurosignaling system is involved in specific sensory mechanisms is to challenge it through pharmacological modulation and observe a change in function (Whelan et al., 2023). We have adopted this approach and previously shown that a single dose of the GABA type B (GABA_B_) receptor agonist arbaclofen (STX209) enhances suppression of cortical electroencephalogram (EEG) responses to sensory stimuli in both the visual and auditory domains in individuals with ASD, but does the opposite in nonautistic people (Huang et al., 2022). This suggests that individuals with ASD have differences in GABAergic function/responsivity as measured using EEG; but these differences are not domain specific.

While these studies have been useful first steps, they have relied upon relatively burdensome and/or expensive designs involving MR spectroscopy and/or EEG which are not suitable for everyone. They also do not tell us whether autistic differences in the GABAergic control of sensory processing exist beyond the cortex. Exploring wider regions beyond the cortex may therefore help us deliver more “simple”, inexpensive tools to objectively assess an individual's GABAergic control of sensory processing. In this study, we examined the potential GABAergic modulation of light processing at the level of the retina in adults with and without ASD. As the retina is part of the central nervous system (CNS) and develops alongside the brain in embryonic life, ASD-associated genetic and/or environmental exposures that modulate neurodevelopment could therefore be hypothesized to influence retinal signaling pathways. The retina is more easily accessible than the brain; thus, capturing GABAergic function via the retina may not only expose additional differences along sensory processing pathways in ASD but may also offer a novel, efficient, and practical opportunity to assess target engagement and improve trial stratification.

The functional integrity of the retina can be conveniently assessed using the ERG with handheld devices (Constable et al., 2020; Lee et al., 2022; Robson et al., 2022). For example, in response to a single flash delivered in photopic conditions, the ERG a-wave captures responses from cone photoreceptors and cone-driven OFF bipolar cells, and the b-wave is generated by simultaneous activity in cone-driven ON and OFF bipolar cells. Additional information can be acquired from the 30 Hz flicker response and the PhNR. The 30 Hz flicker response is driven by cone photoreceptors, but arises from the ON and OFF bipolar cells (Picton et al., 2003; McCulloch et al., 2015). The PhNR is a late negative component following the b-wave, which arises from retinal ganglion cells. It is thought to be best elicited by a red flash delivered on a blue background (Frishman et al., 2018).

Overall, the “vertical” signal transmission from photoreceptors to bipolar cells and ganglion cells is mainly glutamatergic, but there is substantial modification of transmission by lateral interneurons, namely, horizontal cells (which modulate transmission between photoreceptors and bipolar cells) and many types of amacrine cells (mediating transmission between bipolar cells and modifying transmission from bipolar to ganglion cells). Many of these lateral interactions comprise GABAergic inhibition (Yazulla, 1986; Davanger et al., 1991; Yang, 2004). Hence, if E–I alterations in individuals with ASD are generalized across the central nervous system, they may also manifest as functional changes in the retina assessed using the ERG, especially in responses (a- and b-wave) to single flashes as they capture the vertical glutamate pathway from photoreceptors to bipolar cells, and this transmission is expected to be modulated by GABAergic horizontal cells.

Functional differences in the retina have been previously explored in ASD, but results from case–control studies have been inconsistent. For instance, attenuated a- and b-wave amplitudes were reported in children and adolescents with ASD at higher flash strength over 0.95 log phot cd·s·m^−2^ (Constable et al., 2016, 2020). In contrast, others found a small increase in the a- and b-wave amplitudes in adults with ASD (Friedel et al., 2022a). Cohort differences in age and/or intelligence quotient (IQ) may have contributed to the heterogeneity of these findings (Friedel et al., 2022a). Also, to the best of our knowledge, all previous studies were restricted to comparisons of baseline ERG measurements and did not capture the dynamic control of function by the E–I system; and to date, the hypothesis that GABA-dependent regulation of retinal visual processing is altered in ASD has not been directly tested.

Here we carried out a double-blind, placebo-controlled crossover experiment to test the hypothesis that a single oral dose of arbaclofen (STX209) differentially alters the ERG in individuals with and without ASD. Based on our previous studies of cortical EEG responses in the same population (Huang et al., 2022, 2023), we hypothesized that the baseline ERG would be atypical in individuals with ASD and arbaclofen would modulate the retinal response differently between the ASD and TD groups. We additionally explored whether any associations were seen with the flicker ERG or the PhNR.

We also extended this work to understand its broader implications in two ways.

1. We assessed whether the retinal response to arbaclofen correlated with our previously reported cortical responses in the same individuals. If so, this would indicate that the retinal ERG is a potentially useful proxy for cross-domain brain GABA function and target engagement.
2. We explored the relationship of GABAergic retinal response with more complex autistic traits captured using the autism quotient (AQ; Baron-Cohen et al., 2001) across the entire cohort. If an association was found, this would support our proposal that sensory processing differences in autism are upstream of more complex autistic traits and therefore potentially a tractable intervention target than more complex downstream “hard-to-measure” behaviors.

## Materials and Methods

### Study design

This study was implemented as part of a series of experiments to investigate the role of GABA on ASD (Huang et al., 2022) as approved by King's College Research Ethics Committee (RESCM-17/18-4081 and LRS-15/16-3582). The U.K. Medicines and Healthcare products Regulatory Agency (MHRA) confirmed that this design was “not a Clinical Trial of an Investigational Medicinal Product (IMP) as defined by the EU Directive 2001/20/EC and no submission to the Clinical Trials Unit at the MHRA is required.”

Participants were given an oral dose of placebo, 15 or 30 mg of arbaclofen (STX209), in a randomized and double-blind order. ERG was acquired approximately 4 h after drug/placebo administration and within the 5-h half-life of arbaclofen (Sanchez-Ponce et al., 2012). Study visits were at least 1 week apart to ensure complete drug washout. All the tests were conducted at the Institute of Psychiatry, Psychology and Neuroscience, King's College London.

Medical cover was provided throughout each test session, and participants were asked to remain at our unit at least 4 h after drug/placebo intake. The physician was “blind” to the order of administration but had access to the randomization codes held at our pharmacy and by the chief investigator in the event that an emergency code break was required. No emergency code break was required, but where a participant had experienced side effects that were more than moderate in the opinion of the study clinician and after discussion with the chief investigator, unblinding occurred to try to avoid exposure to a higher dose of arbaclofen on a subsequent visit as previously reported (Huang et al., 2022).

### Participants

Sixty-one participants (*n* = 22 ASD) were included in this study. All participants were adults, aged from 19 to 53, with IQ [on the Wechsler Abbreviated Scale of Intelligence II (WASI-II)] >70. Demographic characteristics including biological sex and IQ did not differ between the TD and ASD groups (Table 1). The mean age of the ASD group (36.1 ± 10 years) was higher than that of the TD group (28.6 ± 8.1 years) (*t*_(61) _= 3.1; *p* = 0.003); thus, age was controlled in statistical analyses. There were no correlations between age and ERG outcomes in our cohort. Participants with ASD with a known genetic cause, such as fragile X syndrome, neurofibromatosis type 1, or 22q11 deletion syndrome, were excluded from the study. Other inclusion criteria were as follows: ability to give informed consent, no comorbid psychiatric illness such as psychotic illness and major mood disorder, no history of seizures or diagnosis of epilepsy, and no physical illness, such as heart disease, high blood pressure, and renal insufficiency.

**Table 1.** Participant demographic data and ASD clinical scores

Measure	TD	ASD	Statistic	*P*
Number (male/female)	22/17	16/6	*X*^2^ = 1.6	0.2
Age	28.6 ± 8.1	36.1 ± 10	*t* = 3.1	0.003
Full-scale IQ	119.8 ± 10.1^a^	118.1 ± 10.3^b^	*t* = 0.6	0.5
AQ	16.9 ± 8^c^	36.4 ± 7^d^	*t* = 9.2	1.2 × 10^−12^

Values are shown as mean ± SD. Group difference of age, IQ, and AQ scores tested using independent-sample *t* tests; comparison of proportion of males and females tested using the chi-squared test. In the expert opinion of the team, IQ > 70 was safe to assume in these participants based on education/employment status.

a As a result of Covid lockdown restrictions/participant preference, it was not possible to complete in-person IQ testing on four TDs.

b As a result of Covid lockdown restrictions/participant preference, it was not possible to complete in-person IQ testing on three ASDs.

c AQ data not returned for five TDs.

d AQ data not returned for one ASD.

For the ASD group, we required a clinical diagnosis fulfilling ICD 10 and/or DSM 5 criteria to be in place. ASD traits were assessed across both the TD and ASD groups using the AQ (Baron-Cohen et al., 2001). In the month preceding participation, four nonautistic participants were taking regular medication with drugs such as cetirizine (one), levothyroxine (one), and antihistamine (two); nine autistic participants were taking regular medication with drugs such as sertraline (five), ibuprofen (two), citalopram (one), and cyclizine (one). None of these drugs target glutamate or GABA pathways directly. All other participants were medication-free.

### Data acquisition and protocols

Full-field, right eye ERGs were recorded using the RETeval portable instrument (LKC Technologies). Stimuli were generated by LEDs within the mini-Ganzfeld dome. A sensor strip (LKC Technologies) incorporating skin electrodes (active, reference, and ground electrodes) was placed 2–3 mm below the lower eyelid across the upper portion of the zygomatic process, with the inner border aligned vertically to the center of the pupil. Participants were seated and instructed to look at the center of the Ganzfeld dome and avoid excessive blinking or eye movements. The RETeval worked in the “Troland” mode in which the device continually measured pupil size and adjusted stimulus strength to achieve the desired retinal illuminance. Recordings were automatically stopped if pupil tracking was lost due to poor fixation, pupil diameter was <1.8 mm, or electrode impedance was >5 kΩ. Participants were exposed to ambient room lights (estimated luminance approximately 40 photopic cd m^−2^) for at least 10 min for light adaptation before tests. Three protocols established by the International Society for Clinical Electrophysiology of Vision (ISCEV) were used.

1. ISCEV standard light-adapted 3 ERG

This was our key protocol of interest because the LA3 ERG a- and/or b-wave to single flashes capture the “simple” vertical glutamate pathway from photoreceptors to bipolar cells, and this transmission is expected to be modulated by GABAergic horizontal cells. Specifically, repeated single white flashes (1.96 Hz, 85 Td·s) were presented superimposed on a white background (850 Td). The white stimuli and background (1931 CIE *x*, *y* of 0.33, 0.33) were generated by red, green, and blue LEDs (peak wavelengths of 621, 530, and 470 nm, respectively). The test period was ∼15 s and thus responses to 30 flashes were recorded and averaged.

For completeness, we also included two additional protocols to capture whether arbaclofen modulated subsequent processing of the light signal when it engaged other retinal components, as measured in the ERG.

1. ISCEV standard light-adapted 30 Hz flicker ERG

Quick flickering white stimuli (28.3 Hz, 85 Td·s) were presented superimposed on the white background (850 Td). The test lasted ∼5–15 s and included 141–424 flashes.

1. PhNR 3.4 Hz ERG

Single red flashes (3.4 Hz, 38 Td·s; red LED peak wavelength of 621 nm) were presented superimposed on a rod-saturating blue background (380 Td; blue LED peak wavelength of 470 nm). The test lasted ∼60 s and included 200 flashes. This protocol uses red flashes not only to evoke an a- and b-wave but also to enhance the PhNR component which occurs after the b-wave and is related to retinal ganglion cell activity.

### ERG analyses

Each test was performed twice (two trials) in a participant visit. Raw ERG signals were processed by the in-built algorithm for each protocol. The reliability of reported responses was manually checked blind to group and drug/placebo condition by experienced clinical scientists (S.M.L. and O.A.M.). Unreliable ERGs showing drift, noise, or poor reproducibility were excluded. The outcome was averaged across the two repeated trials if both were available and survived the reliability check. When only one trial survived, this single trace was used for that participant.

We evaluated the a- and b-wave amplitudes and their peak times for the ISCEV standard LA 3 ERG test (Protocol 1) and the PhNR 3.4 Hz Td ERG test (Protocol 3). We assessed the maximal amplitude and the peak time for the ISCEV standard LA 30 Hz flicker ERG test (Protocol 2). For Protocol 3, following published methods (McCulloch et al., 2015; Robson et al., 2022), the PhNR amplitude at 72 ms (*p*_72_) (Preiser et al., 2013) and at the minimum between 55 and 100 ms (*p*_min_) and the time to the minimum were also assessed. Two PhNR-related ratios, the *P*-ratio (Preiser et al., 2013) and the *W*-ratio (Mortlock et al., 2010), were calculated as follows (*b* and *a* refer to the b-wave and a-wave amplitudes, respectively):

(1) $$P_{\mathrm{ratio}}=p_{72}/b,$$

(2) $$W_{\mathrm{ratio}}=(b-p_{\min})/(b-a),$$

### Post hoc analyses

Our main results for group differences and drug regulation effect were identified in the a-wave responses to flash stimulation (see Results); therefore, we tested the hypothesis that the retinal a-wave responses to arbaclofen would be correlated with their cortical responses to the drug indexed by EEG as previously reported (Huang et al., 2023). We also tested whether an individual's a-wave responses to arbaclofen would be correlated with the extent of their autistic traits measured by total AQ scores. Specifically, we defined an individual “sensitivity index” as the placebo–30 mg difference (shift) in the a-wave amplitude to single white flashes in 29 participants (*n* = 14 ASD) who had completed both the placebo and 30 mg visits and examined the correlation between the sensitivity index and AQ across the whole cohort.

### Previous cortical tests

#### Visual cortical test

Steady-state visually evoked potentials (SSVEP) were induced by a visual contrast sensitivity paradigm comprising two parts: (1) four flickering circular foreground gratings with sinusoidal vertical Gabor grating patterns (1 cycle per degree, radius of 2°) and (2) a static background which may (vertical grating) or may not (blank) interfere. In ASD, results from this task indicated that, at baseline, the occipital cortex is more responsive in conditions of low foreground contrast with interference from the background and less responsive when the foreground contrast is high and the background interference is low. GABA_B_ receptor activation made the response in autistic participants comparable to nonautistic participants at baseline (i.e., responses increased with higher foreground contrast and less background interference. To capture this “shift,” we calculated an individual “sensitivity index” to arbaclofen for each participant (Huang et al., 2022).

#### Auditory cortical test

Mismatch negativity and repetition suppression are measured with an oddball paradigm. The stimulus train comprised sinusoidal tones: 82% standard trials (1,000 Hz, 50 ms), 6% frequency deviants (1,200 Hz, 50 ms), 6% duration deviants (1,000 Hz, 100 ms), and 6% combined frequency-duration deviants (1,200 Hz, 100 ms) in random order. We reported less suppression of repetitive auditory stimulation in autism than nonautistic individuals, and demonstrated that GABA_B_ agonism with arbaclofen strengthened (weaker) auditory suppression in autistic individuals, but disrupted (intact) auditory suppression in neurotypical individuals. Thus, this study provided evidence that GABAergic regulation of auditory processing is fundamentally different in autism (Huang et al., 2023).

### Statistical analysis

Independent-sample *t* tests were used to assess ASD–TD group differences in each of the ERG parameters described above. The placebo–30 mg shift difference in the a-wave amplitude was assessed using a paired *t* test. For correlation analysis, the reported *r* values are Pearson's linear correlation coefficients; associated *p* values were computed using a Student's *t* distribution for a transformation of the correlation. Statistical thresholds were set at *p* < 0.05. Two-tailed and statistical significances were corrected for multiple comparisons using the Benjamini–Hochberg method (Benjamini and Hochberg, 1995). The Shapiro–Wilk test was used to check normality and the data were found to not differ significantly from normal distributions.

The overall drug modulation and group effect on each of the ERG parameters were assessed using a linear mixed-effect model (LMM). The model was built with group and drug dose as fixed-effects variables and with the group–drug interaction included. When a significant interaction was observed, the LMM shrank to be a simple linear model with drug dose as the independent variable and was separately applied to TD and ASD to measure the within-group drug effect. For our main result (the a-wave amplitude differentially shifted by arbaclofen in ASD and TD groups), the LMM procedure was repeated while controlling for age, gender, and IQ.

## Results

Demographics of participants are shown in Table 1. Thirty-nine TD (21 males, 18 females) and 22 ASD (16 males, 6 females) were recruited and 134 study visits were completed. Seven visits were excluded after the reliability check showing drift, noise, or poor reproducibility. The remaining visits were divided into subgroups by group and drug/placebo condition: 45 placebo (P) visits (*n* = 18 ASD), 45 low-dose (L) visits (*n* = 16 ASD), and 37 high-dose (H) visits (*n* = 14 ASD). The null hypothesis held for the normality check of our main outcomes using the Shapiro–Wilk test.

### ISCEV standard LA 3 ERG

**Figure 1.**
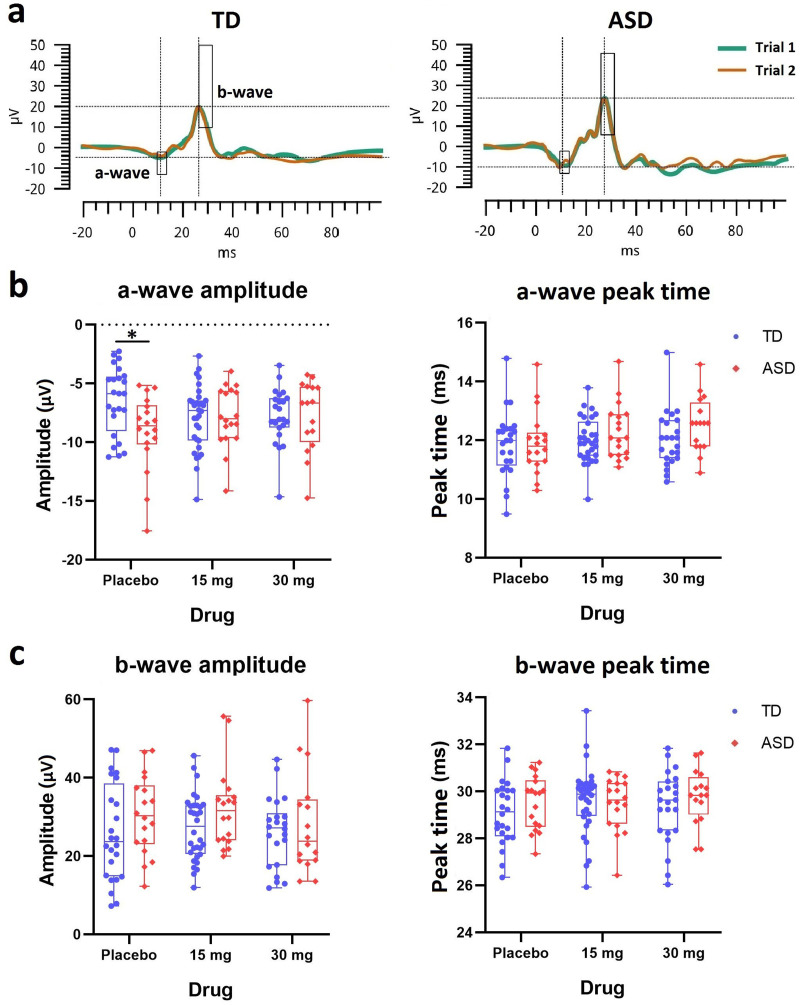
ERG waveforms and parameters in response to single white flashes. ***a***, Exemplary representation of the ERG waveforms at baseline for a nonautistic participant and an autistic participant. ***b***, Scatter plots of the amplitude (µV) and peak time (ms) of the a-wave for TD (blue) and ASD (red). ***c***, Scatter plots of the amplitude (µV) and peak time (ms) of the b-wave for TD (blue) and ASD (red). *, the ASD–TD group difference is significant; corrected *p* < 0.05.

Sample ERG waveforms in response to ISCEV standard single white flashes are shown in Figure 1*a*. The a-wave appeared as a negative trough at ∼12 ms after the stimulus onset; the b-wave was a positive peak following the a-wave at ∼30 ms. Scatter plots of parameters (amplitude and peak time) of a-wave and b-wave are shown in Figure 1*b,c*, respectively. At placebo, the a-wave amplitude in participants in the ASD group (mean = −9.0 µV; SD = 3.2) was more prominent than that recorded from participants in the TD group (mean = −6.4 µV; SD = 2.9), resulting in a significant group difference (*t*_(40) _= 2.8; *p* = 0.03). The baseline difference in the a-wave amplitude was abolished by arbaclofen administration at both 15 mg and 30 mg. This was confirmed by LMM results showing there was a significant drug–group interaction (*t*_(127)_ = 2.8; *p* = 0.02). Specifically, arbaclofen was associated with decreased a-wave amplitude in ASD, making it more similar to measures in the TD group at baseline (eff = −0.7; *t*_(77)_ = −2.4; *p* = 0.02). In contrast, arbaclofen tended to increase the a-wave amplitude in the TD group, making it more similar to the measures in the ASD group at baseline; however, this result did not reach statistical significance (eff = 0.7; *t*_(50)_ = 1.7; *p* = 0.1).

**Figure 2.**
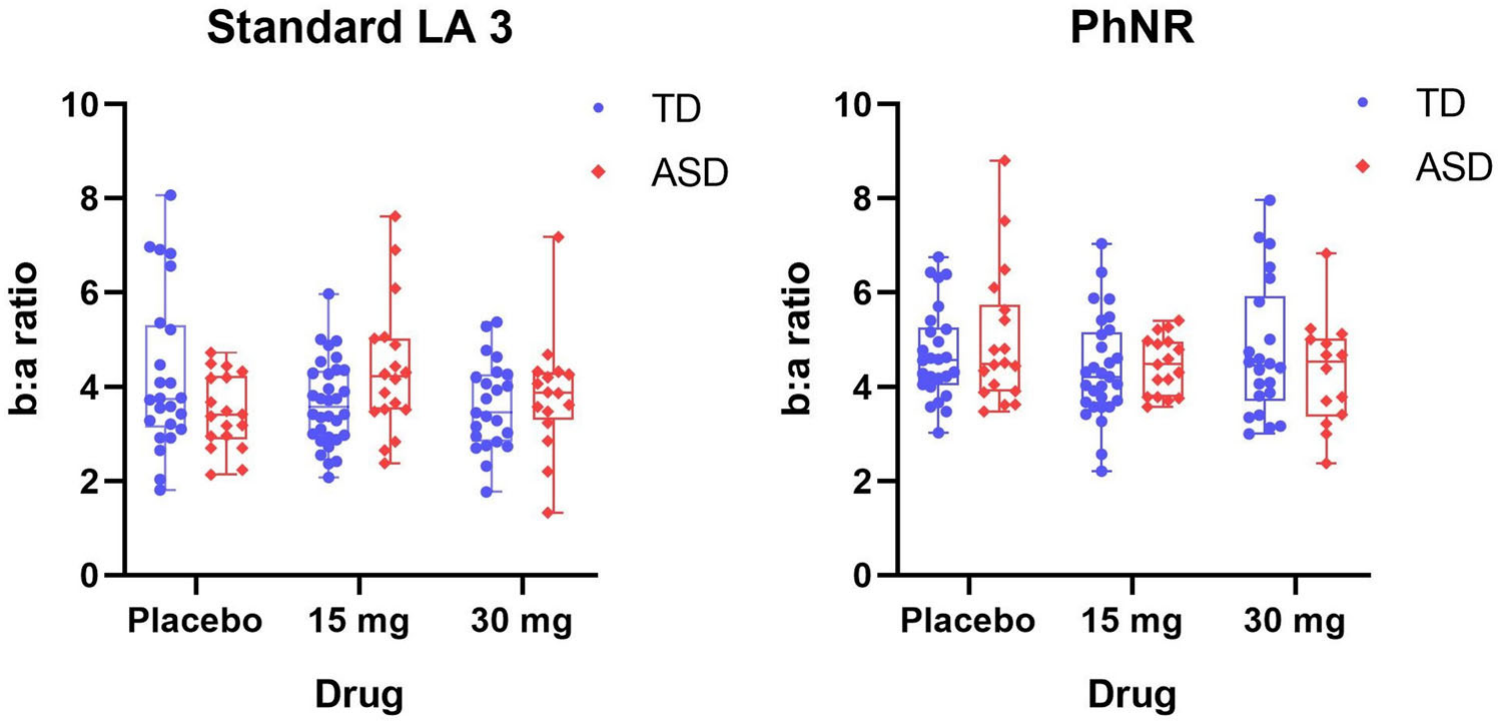
The scatter plots of b:a ratio. Under the standard LA 3 condition, the b:a ratio tended to be larger in TD relative to ASD (*t*_(40) _= 1.8; *p* = 0.07), and this tendency was potentially regulated by the 30 mg administration (*t*_(37) _= 0.6; *p* = 0.55). There were no group differences or drug modulation effect observed at the PhNR protocol after correction for multiple comparisons.

The baseline b-wave amplitude in ASD (mean = 30.6 µV; SD = 9.9) was also larger than in TD (mean = 25.9 µV; SD = 12.4), but the difference was not significant (*t*_(40) _= 1.3; *p* = 0.2). There was no drug–group interaction or any drug effects observed in the LMM for the b-wave amplitude. Moreover, there were no group differences or drug effects observed for any peak time parameters. No group differences or drug effects were observed in the b:a ratio (Fig. 2), suggesting that the b-wave was also larger in participants with larger a-waves. Thus, the autistic retina was “over-responsive” to single white flashes; GABA_B_ agonism was associated with suppressed retinal responses in ASD but tended to do the opposite in TD.

### 30 Hz flicker ERG

**Figure 3.**
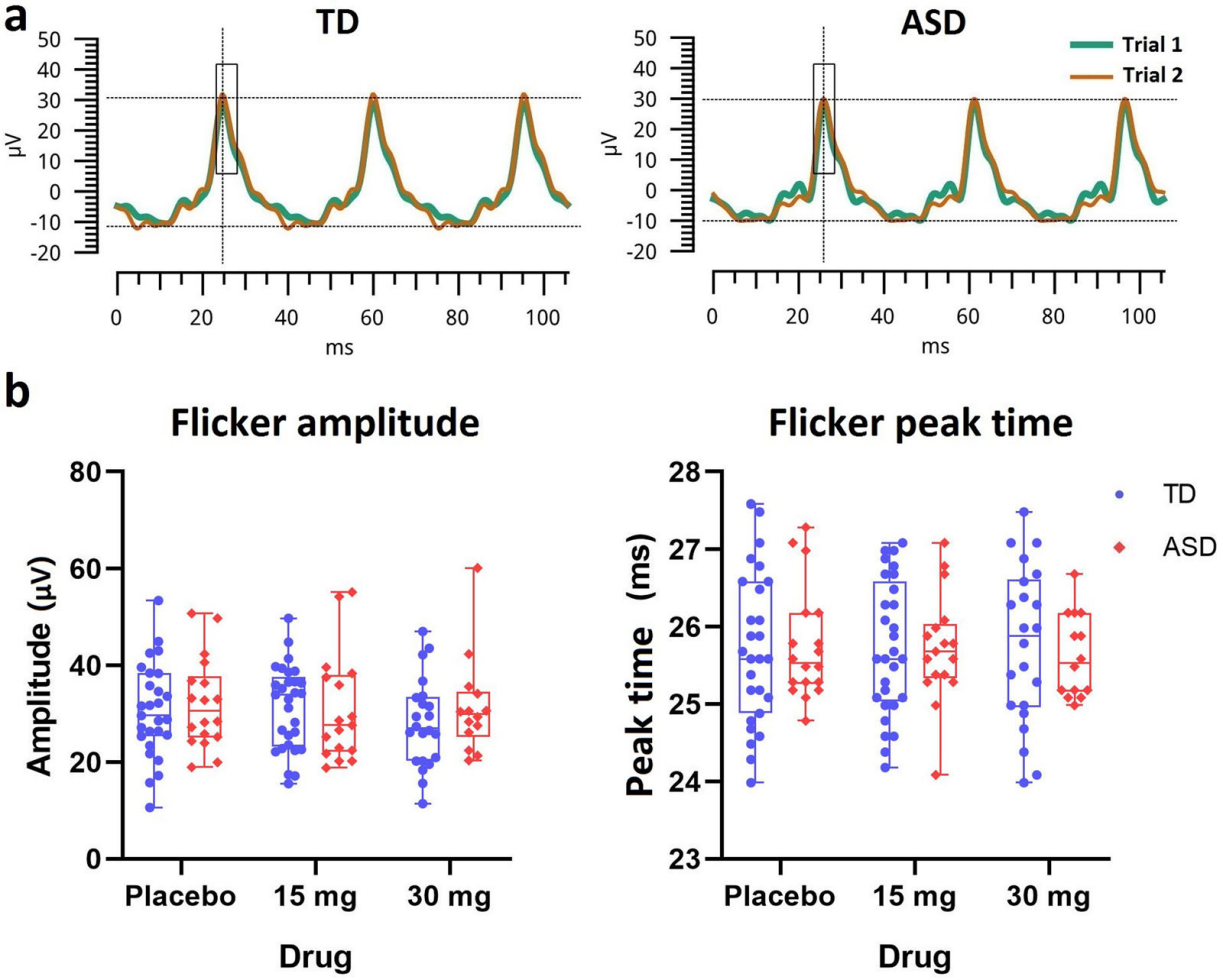
ERG waveforms and parameters in response to the 30 Hz flicker stimulus. ***a***, Exemplary representation of the ERG waveforms at baseline for TD and ASD. ***b***, Scatter plots of the flicker amplitude (µV) and peak time (ms) for TD (blue) and ASD (red).

Sample ERG waveforms in response to ISCEV standard-equivalent 30 Hz flicker stimuli are shown in Figure 3*a*. Scatter plots of flicker response parameters are shown in Figure 3*b*. The peak amplitude and the peak time were compared and no group differences were observed. At placebo, the peak amplitude was comparable between ASD and TD groups (mean_ASD_ = 31.1 µV; SD_ASD_ = 9.3; mean_TD_ = 29.7 µV; SD_TD_ = 9.6; *t*_(43)_ = −0.5; *p* = 0.6). The baseline peak time was also comparable between the two groups (mean_ASD_ = 25.8 ms; SD_ASD_ = 0.7; mean_TD_ = 25.7 ms; SD_TD_ = 1.0; *t*_(43)_ = −0.1; *p* = 0.9). No interaction or drug effect was observed by LMM results for either amplitudes or peak times.

### PhNR 3.4 Hz ERG

**Figure 4.**
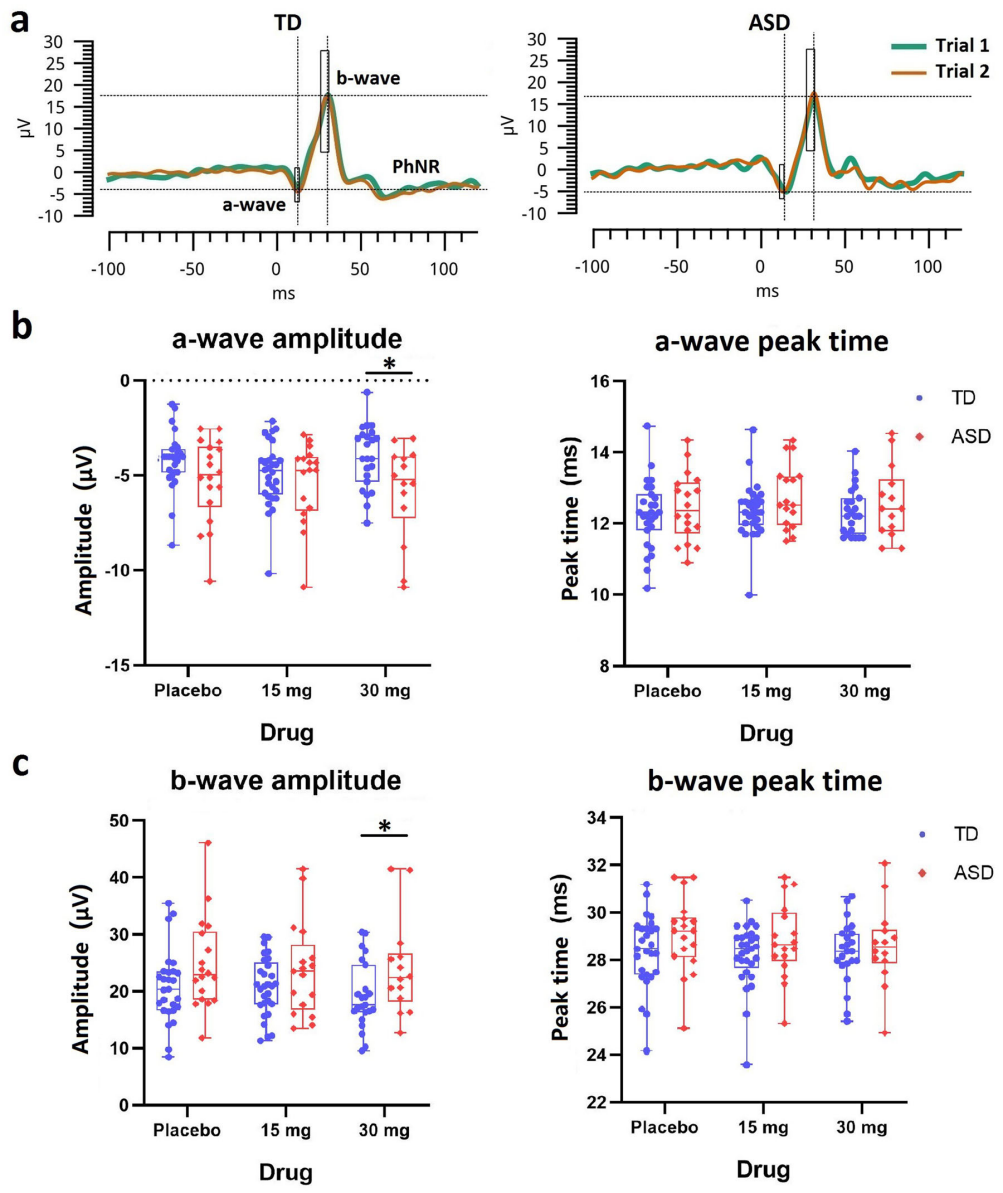
ERG waveforms and parameters for the PhNR stimulation protocol. ***a***, Exemplary representation of the ERG waveforms at baseline in response to red flashes with blue background for a nonautistic participant and an autistic participant. ***b***, Scatter plots of the amplitude (µV) and peak time (ms) of the a-wave for TD (blue) and ASD (red). ***c***, Scatter plots of the amplitude (µV) and peak time (ms) of the b-wave for TD (blue) and ASD (red). *, the ASD–TD group difference is significant; corrected *p* < 0.05.

The PhNR-eliciting red flashes evoked a negative a-wave at ∼12 ms and a positive b-wave at ∼30 ms after stimulus onset (Fig. 4*a*). At placebo, similar to their responses to standard white flashes, participants with ASD had a greater a-wave amplitude compared with TD participants, but this was not statistically significant (*t*_(42)_ = 1.8; *p* = 0.16; Fig. 4*b*). However, there was a significant group difference at 30 mg arbaclofen with a larger a-wave amplitude in the ASD group (*t*_(35)_ = 2.6; *p* = 0.04). The baseline b-wave amplitude in ASD also tended to be larger than that in TD (*t*_(42)_ = −1.8; *p* = 0.16; Fig. 4*c*), and this apparent autistic overactivity was sustained at 30 mg arbaclofen (*t*_(35)_ = −2; *p* = 0.08). LMM results confirmed an overall group difference in the b-wave amplitude (eff = 4.1; *t*_(125)_ = 2.5; *p* = 0.04), with no drug effect or drug–group interaction observed. No group differences, drug effect, or interaction were observed for any peak time parameters. There was no correlation between the a-wave amplitudes in response to standard white flashes and red-on-blue PhNR flashes in ASD (*r* = 0.2; *p* = 0.44).

The PhNR component is an additional negative trough following the b-wave (Fig. 4*a*). There were no group differences, drug effect, or interaction observed in any of the five PhNR-related parameters (*p*_72_, *p*_min_, time to *p*_min_, *P*-ratio, and *W*-ratio; see Materials and Methods). Placed together, these results indicate a trend toward a larger a- and b-wave amplitudes in ASD in the PhNR test which are not markedly modified by GABA agonism.

### Relations to cortical responses and wider autistic symptoms

**Figure 5.**
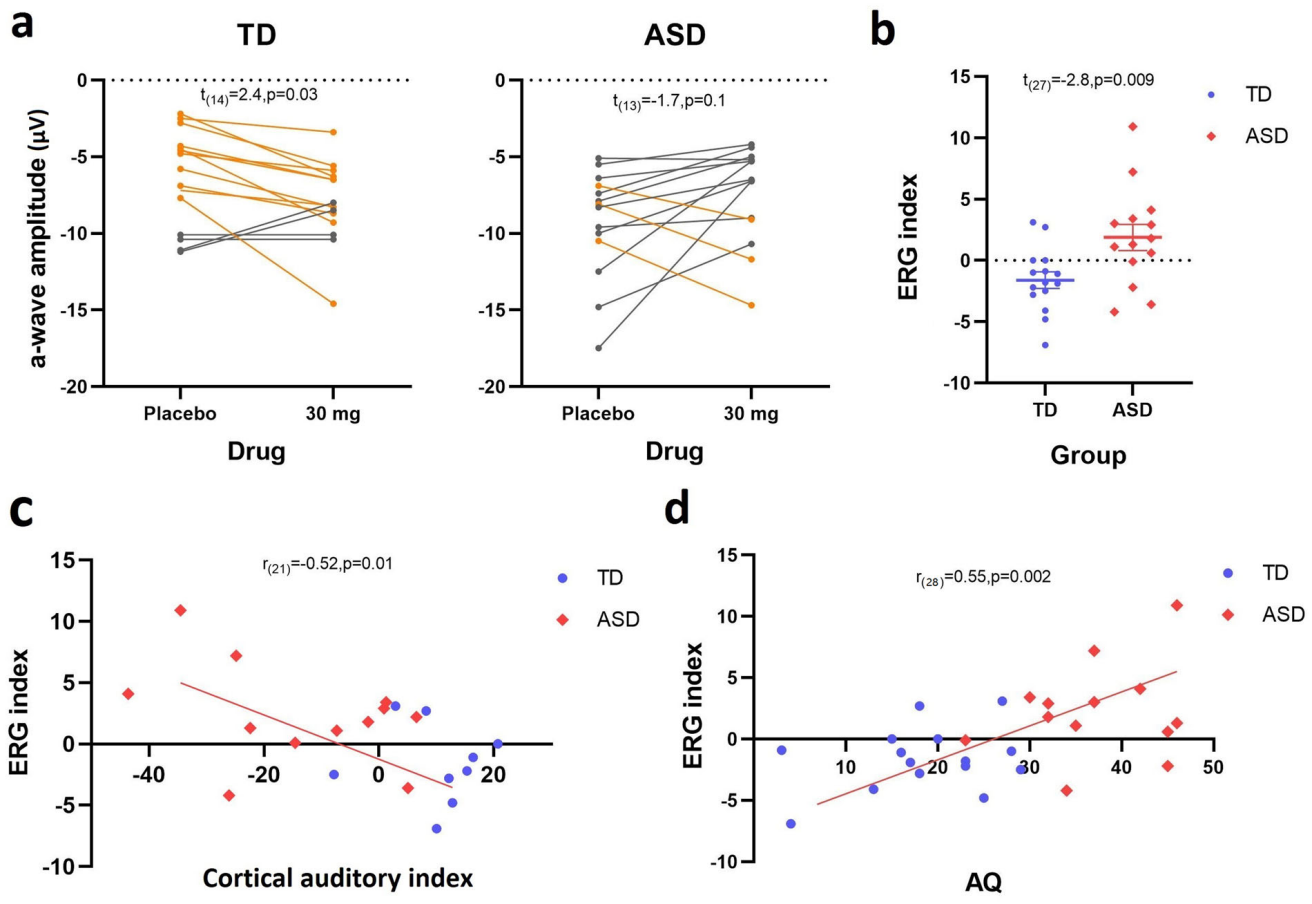
Retinal sensitivity index and relationship with cortical responses and wider autistic traits. ***a***, The transition graphs to show the shift effect between placebo and 30 mg arbaclofen on TD and ASD. Yellow lines indicate an increase in the a-wave amplitude to white flashes following arbaclofen (the majority of the TD group); gray lines indicate a decrease in the a-wave amplitude following arbaclofen (the majority of the ASD group). ***b***, Scatter plots of extracted ERG sensitivity indices. ***c***, The negative correlation observed between the retinal sensitivity index and previously reported cortical responses to arbaclofen indexed by EEG. ***d***, The positive correlation observed between the retinal sensitivity index and total AQ scores.

Our main finding was as hypothesized—a differential shift in the a-wave amplitude elicited by single flashes in response to arbaclofen in ASD and TD groups (Fig. 5*a*). Therefore, using our previously reported approach (Huang et al., 2022), the difference in the a-wave amplitude between placebo and 30 mg arbaclofen was calculated as a “sensitivity index” to GABA agonism, and a significant group difference was confirmed (*t*_(27)_ = −2.8; *p* = 0.009; Fig. 5*b*).

We then tested the hypothesis that retinal responses to arbaclofen would be correlated with our previously reported cortical responses to arbaclofen in the same individuals (Huang et al., 2022). As predicted, the retinal sensitivity index derived from the a-wave amplitude had a strong negative correlation with cortical auditory responses to arbaclofen indexed by EEG (*r*_(21)_ = −0.52; *p* = 0.01; Fig. 5*c*). The retinal sensitivity index also tended to be correlated with cortical visual-evoked potentials in response to arbaclofen, though this did not reach statistical significance (*r*_(11)_ = 0.55; *p* = 0.08), potentially due to the small sample size of individuals with overlap between the retinal and visual tests.

We also tested whether, across all subjects, retinal responses to arbaclofen would be correlated with the extent of their autistic traits as measured by AQ scores. As predicted, there was a significant positive correlation between the retinal sensitivity index and total AQ scores across the whole cohort (*r*_(28)_ = 0.55; *p* = 0.002; Fig. 5*d*). No correlation between the sensitivity index and age or IQ was observed. Thus, the more atypical an individual's GABA_B_-dependent retinal dynamics were, the more autistic traits (higher total AQ scores) they had.

## Discussion

Differences in sensory processing are core to ASD, and these differences have been postulated to arise from GABAergic dysfunctions in the central nervous system (Robertson and Baron-Cohen, 2017). The retina has a well-characterized neural structure with similar foundations to other regions of the CNS. Preclinical studies indicate that lateral GABA neurons are involved in modulating vertical glutamate signal transmission between retinal neurons (Yang, 2004; Hirano et al., 2020). However, to date, the hypothesis that GABA-dependent regulation of retinal visual processing as measured by the ERG is altered in ASD has not been directly tested.

In this study, we combined pharmacological challenge with the GABA_B_ receptor agonist arbaclofen and light-adapted ERGs evoked by three protocols designed to test retinal responses to single white flashes, flicker, and single red flashes, respectively. We observed that the a-wave amplitudes in response to single white flashes were more prominent in ASD at baseline (placebo), relative to those in TD; and this difference was modulated by targeting GABA_B_. Arbaclofen was associated with a decrease in the a-wave amplitude in ASD, but an increase in TD, eliminating the baseline group difference. We also discovered that at an individual-level greater shift in retinal responses to arbaclofen was associated with more prominent cortical EEG responses and more autistic characteristics as measured by total AQ scores in the same cohort. A trend toward increased responsivity in ASD was also observed in the a- and b-wave amplitudes to single red flashes under the PhNR protocol. Taken together, our results are consistent with the notion that individuals with ASD may have baseline retinal overactivity to repetitive single light flashes and that the GABAergic system modulates this retinal response differently in autistic and nonautistic individuals.

We elected to use the AQ based on evidence that autistic traits are continuously distributed across the population (Wing, 1988; Constantino et al., 2003; Posserud et al., 2006) and the AQ is intended to capture autistic traits across a neurodiverse population (autistic and nonautistic individuals) (Ruzich et al., 2015). In this study, we used the AQ to understand if GABAergic retinal responses were related to the extent of autistic features across the group rather than to support diagnoses. There are of course alternatives, for example, the Autism Diagnostic Observation Schedule (ADOS) (Pruette, 2013). However, the ADOS is not easily used in nonautistic people and the standardized ADOS scores in the modest sample size of autistic participants in this study would have precluded meaningful correlational analyses and insights into the TD group.

**Figure 6.**
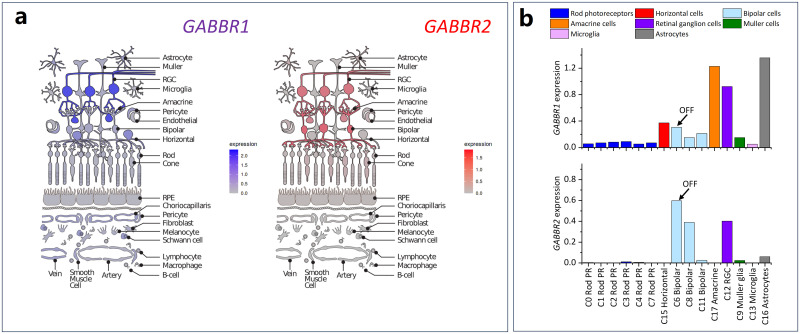
GABA_B_ receptor expression in the retina. Arbaclofen may act on the GABA_B_ receptor subunit 1 (GABBR1) or 2 (GABBR2) targets in the retina. ***a***, Schematics showing expression of each gene by broad cell type, generated from the “Spectacle” resource (https://singlecell-eye.org/app/spectacle/ accessed 18 Nov. 2023), using the “IOWA-integrated retina, RPE, and choroid” dataset (Voigt et al., 2020). ***b***, Data plotted from a supplementary table of a published study from three human donors (Lukowski et al., 2019). Eighteen transcriptionally distinct clusters (C0–C17) were reported. Data plotted here are for expression in 15 clusters [data for the following clusters are not shown: C10 (cone photoreceptors) had minimal expression; C5 (others) and C14 (others) are also omitted, as the cell type was not identifiable]. The C6 (bipolar) cluster (bar labeled “OFF”) was identified as representing OFF bipolar cells due to high expression of GRIK1. PR, photoreceptor; RGC, retinal ganglion cell; RPE, retinal pigment epithelium.

Light-adapted ERG components have distinct cellular origins along the “vertical” pathway in the retina—from photoreceptors to bipolar cells to retinal ganglion cells (Euler et al., 2014). This “vertical” signal transmission is modulated by two types of interneurons, horizontal cells and amacrine cells, modifying transmission in the outer and inner plexiform layers (OPL, IPL), respectively (Yang, 2004). Much of this involves GABAergic transmission. Horizontal cells provide feedback inhibition to cone photoreceptors and feedforward inhibition to bipolar cell dendrites through GABA_B_ and other GABA receptor subtypes (Wu, 1991; Yang and Wu, 1991). Thus, a candidate mechanism underpinning the observed larger baseline a-wave amplitude in ASD might be dampened inhibitory interaction of horizontal cells with cone-driven OFF bipolar cells, which was reversed by boosting GABAergic function through arbaclofen. It is also of interest that altered retinal layer volumes have been reported in individuals with ASD (García-Medina et al., 2017; Friedel et al., 2022b). Figure 6 depicts data derived from single-cell expression studies of the human retina (Lukowski et al., 2019; Voigt et al., 2020), showing the retinal cell types that express genes for GABA_B_ receptor subunits and whose activity might therefore be affected by arbaclofen. Both receptor subunits are expressed in bipolar cells. Interestingly, of bipolar cell classes, the highest expression is seen in OFF bipolar cells (for *GABBR2*, this is the most highly expressing cell type of all retinal neurons); OFF bipolar cell signals generate much of the light-adapted flash a-wave.

In our results, the baseline group difference was only observed in the a-wave to single white flashes, with no significant difference in the b-wave to the same stimuli. Unlike the a-wave, which essentially arises from the hyperpolarization of cone photoreceptors and OFF bipolar cells, the b-wave is shaped largely by the depolarization of ON bipolar cells, as well as a contribution from recovery from hyperpolarization in OFF bipolar cells. It is possible that an increased a-wave amplitude could result from a delay in onset of the signals generating the b-wave. However, we might then expect the a-wave (and potentially the b-wave) peak times to also be delayed, and in our study, we found no significant difference in peak times between groups. The absence of an effect on the b-wave might reflect more complex modulation of both ON and OFF signals such that any effects on the b-wave cancel each other electrically (Masu et al., 1995; Thoreson and Mangel, 2012). As a result, the measurement of combined ON and OFF bipolar cell responses (the b-wave) might be a less sensitive tool to capture GABAergic challenges.

There was no group difference or modulation effect observed in the flicker responses. The lack of a group difference is consistent with results from a study (Constable et al., 2016) in a smaller sample of similar age, where no group difference was found both for light-adapted 30 Hz flicker responses and dark-adapted 15 Hz flicker responses. A relatively normal flicker response in ASD could be interpreted as likely unaffected calcium gap junction communication between cones and rods in ASD (Constable et al., 2016). 30 Hz flicker responses are cone-driven, but arise in ON and OFF bipolar cells, thus having similar generators to the light-adapted b-wave. As discussed in relation to the flash b-wave, the lack of an observed difference might reflect less effect on ON signals relative to OFF, or effects on both that electrically cancel. Alternatively, it is possible that the steady-state flicker response behaves differently from the transient single-flash response (including in terms of GABAergic inhibition). The steady-state responses evoked by fast and periodic stimuli (like flicker) are commonly thought to reflect brain functions that are independent of transient responses to a slow single event (like a flash) (Stapells et al., 1984; Picton et al., 2003). Although event-related transient responses may predict steady-state responses (Capilla et al., 2011), more evidence shows that the latter cannot be fully explained by the temporal superposition of transient responses and some distinct mechanisms required for fast and sustained synchronization (at specific frequency) of neurons are believed to be involved (Azzena et al., 1995; Conti et al., 1999; Plourde, 2006). Although a potential involvement of GABA in the generation of the retinal flicker responses was implied in previous work, the picture remains complicated (Moskowitz et al., 2012). Thus, we held no a priori hypothesis for the effect of arbaclofen on this signal. Taken together, however, while we are cautious that this may be a type II error, our results suggest there might be limited GABAergic modulation of flicker response pathways in either group.

We also observed a “hyper-responsivity” in the a- and b-wave to single red flashes under the PhNR protocol in the autistic group, but only at trend level of significance. Cones (and, as a consequence, bipolar cell pathways) have specific wavelength sensitivity profiles—cellular responses depend on the stimulus wavelengths (Ammerüller et al., 1995; Roorda and Williams, 1999). Color opponent pathways arise from the bipolar cells onward. Thus, responses evoked by differentially stimulating particular classes of cone (here, the L-cones more than M-cones) may not behave in the same way, including in terms of GABA modulation, as white stimuli that are presumed to stimulate all classes equally. As well as delivering lower retinal illuminance (the photopic strength of the red flashes and blue background were both around half the strength of the white flashes and background), the red flashes (621 nm) would be estimated, based on published spectral sensitivities (Stockman and Sharpe, 2000), to stimulate the L-cones around five times more strongly than the M-cones, with minimal S-cone stimulation. Another possible explanation is that red-on-blue flashes in the PhNR protocol triggered different ON and/or OFF inputs compared with the standard white flashes and hence the differential a-wave and b-wave responses driven by L- and M-cones (Kremers et al., 2021). Moreover, there was no group difference or drug effect observed in any PhNR-related parameters, which is consistent with reports from other groups (Constable et al., 2021; Friedel et al., 2022a). A possible explanation is the multireceptor GABAergic regulation in retinal ganglion cells. Two subtypes of GABA_B_ receptors have been reported in ganglion cells, both of which are sensitive to the parent compound baclofen, but exhibit opposing effects (one excitatory and one inhibitory) on the cell (Shen and Slaughter, 2001). Thus, boosting GABAergic function through arbaclofen might affect competing mechanisms in ganglion cells and give rise to the apparent null effects in the PhNR-related results.

It was beyond the scope of our study to examine how GABAergic modulation alters sensory perception in ASD; however, the strong correlations between the ERG sensitivity index derived here and previous indices derived from our work examining GABAergic responsivity in cortical EEG recordings in response to auditory stimuli point to a generalized GABAergic functional difference across the autistic brain. Moreover, AQ scores and ERG measures were correlated. This supports the concept that the neuropathology underpinning atypical sensory processing links to wider autistic phenotype. As arbaclofen is being considered as a potential intervention option for ASD (Veenstra-VanderWeele et al., 2017), our study raises the possibility that the ERG rapidly acquired using a handheld device could provide a pragmatic stratification tool to establish likely biological response at individual-level pretrial.

This study had limitations: our participant cohort comprised solely of adults (given the ethical constraints of experimental pharmacochallenge studies in children), and thus we cannot determine whether the differences in ERG responsivity to GABA_B_ challenge vary with development. Prior studies have reported an attenuated b-wave and normal a-wave in children and adolescents with ASD (Constable et al., 2016, 2020), which appear in contrast with our findings in adults. A recent study also in an adult cohort reported a slightly larger a- and b-wave in people with ASD relative to nonautistic participants and proposed that cohort differences in age and/or IQ might contribute to the heterogeneity of responses (Friedel et al., 2022a). Thus, although there were no correlations between age and ERG outcomes in our cohort, it is possible that changes in the developmental trajectories of individual participants lead to the diverging results across studies. However, we emphasize that the aim of our work was to examine responsivity rather than the baseline differences. There is already significant heterogeneity in these retinal responses in the general population at baseline, and our hypothesis was that responsivity would depend upon GABA.

For pragmatic reasons, we limited our comparisons to conventionally measured photopic ERG component amplitudes and peak times. It would be of interest in the future to also explore, quantitatively and in a larger sample, whether more subtle aspects of waveform shape (such as oscillatory potential kinetics or time-integrated parameters) vary between groups. Also potentially of note, there is evidence that iris pigment also can alter ERG amplitude (Al Abdlseaed et al., 2010). While theoretically the image gray scale can be used as a coarse proxy for pigmentation and so accommodated in analyses, because one of our goals was to offer a tool that could be used with relative ease (without additional computational demand) as a proxy for the GABA phenotype of individuals with ASD (e.g., prior to clinical trial with GABA-acting drug candidates), we did not pursue this further. However, an advantage of our study was that it was a paired design. Thus, when assessing the effect of arbaclofen relative to placebo, effectively each participant acted as their own control and any contribution of pigmentation should be controlled for.

Finally, we emphasize that the present study does not address the clinical efficacy of arbaclofen as a pharmacological intervention option for ASD, but uses the drug to investigate whether there are GABAergic differences in retinal processing in ASD. Although we observed no statistical effect of sex in response to arbaclofen, given our sample size, it will be important to explore potential sex differences in the future especially as sex differences in sensory processing have been reported in individuals with ASD (Osório et al., 2021).

## Conclusion

Our results suggest that adult autistic individuals have baseline retinal hyper-responsivity to single light flashes and that the GABAergic system modulates this retinal response differently in autistic and nonautistic individuals, removing group differences. The extent of retinal response difference correlated with autistic symptomatology. Placed together with our previous findings, our work suggests that GABA-dependent differences in sensory processes may be “upstream” of more complex autistic phenotypes. Finally, as arbaclofen is being considered as a potential intervention option for ASD, our study raises the possibility that the ERG (which can be rapidly acquired using a handheld device) could provide a pragmatic tool to help stratify individuals using their biological response pretrial. Further studies are required to determine if our findings are replicated and to evaluate their utility in trial settings.
